# Supplementary material for: Metabolite Profiling and Bioassay-Guided Fractionation of Zataria multiflora Boiss. Hydroethanolic Leaf Extracts for Identification of Broad-Spectrum Pre and Postharvest Antifungal Agents
Source: Molecules. 2022 Dec 14;27(24):8903. doi: 10.3390/molecules27248903 (PMC9785509; doi:10.3390/molecules27248903)
Supplement: Supplementary file 1 [file molecules-27-08903-s001.zip › Supplementary Figures and Tables.pdf]

# **Metabolite profiling and bioassay-guided fractionation of *Zataria multiflora* Boiss. hydroethanolic leaf extracts for identification of broad-spectrum pre and postharvest antifungal agents**

Ali Karimi[1,2,*], Torsten Meiners[1], Christoph Böttcher[1]

[1] Institute for Ecological Chemistry, Plant Analysis and Stored Product Protection, Julius Kuehn Institute, Koenigin-Luise-Straße 19, 14195 Berlin, Germany

[2] Institute of Pharmacy, Freie Universitaet Berlin, Koenigin-Luise-Straße 2-4, 14195 Berlin, Germany

* Corresponding author: Ali Karimi

E-Mail: ali.karimi@julius-kuehn.de

**Abstract**

Hydroethanolic leaf extracts of 14 Iranian *Zataria multiflora* Boiss. populations were screened for their antifungal activity against five plant pathogenic fungi and metabolically profiled using a non-targeted workflow based on UHPLC/ESI-QTOFMS. Detailed tandem mass-spectrometric analyses of one of the most active hydroethanolic leaf extracts led to the annotation of 68 non-volatile semi-polar secondary metabolites, including 33 flavonoids, 9 hydroxycinnamic acid derivatives, 14 terpenoids, and 12 other metabolites. Rank correlation analyses using the abundances of the annotated metabolites in crude leaf extracts and their antifungal activity revealed four *O*-methylated flavones, two flavanones, two dihydroflavonols, five thymohydroquinone glycoconjugates, and five putative phenolic diterpenoids as putative antifungal metabolites. After bioassay-guided fractionation, a number of mono-, di- and tri-*O*-methylated flavones as well as three of unidentified phenolic diterpenoids were found in the most active subfractions. These metabolites are promising candidates for the development of new natural fungicides for the protection of agro-food crops.

**Keywords**: Bioassay-guided fractionation; Flavonoids; Hydroxycinnamic acid derivatives; Lamiaceae; Metabolite profiling; Phytopathogenic fungi; Terpenoids; *Zataria multiflora* Boiss..

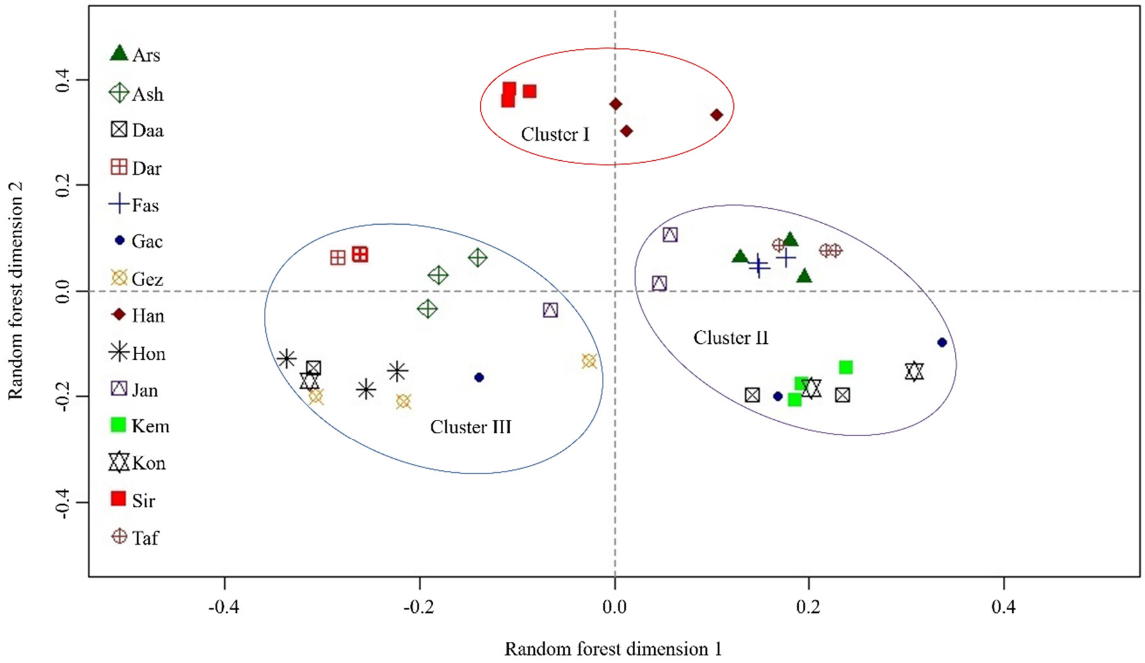


**Figure S1.** Unsupervised random forest classification of metabolite profiles obtained from hydroethanolic *Z. multiflora* leaf extracts (14 populations × 3 samples) by reversed-phase UHPLC/DAD/ESI-QTOFMS operated in negative ion mode. The scatter plot obtained from multidimensional scaling of the proximity matrix is shown.

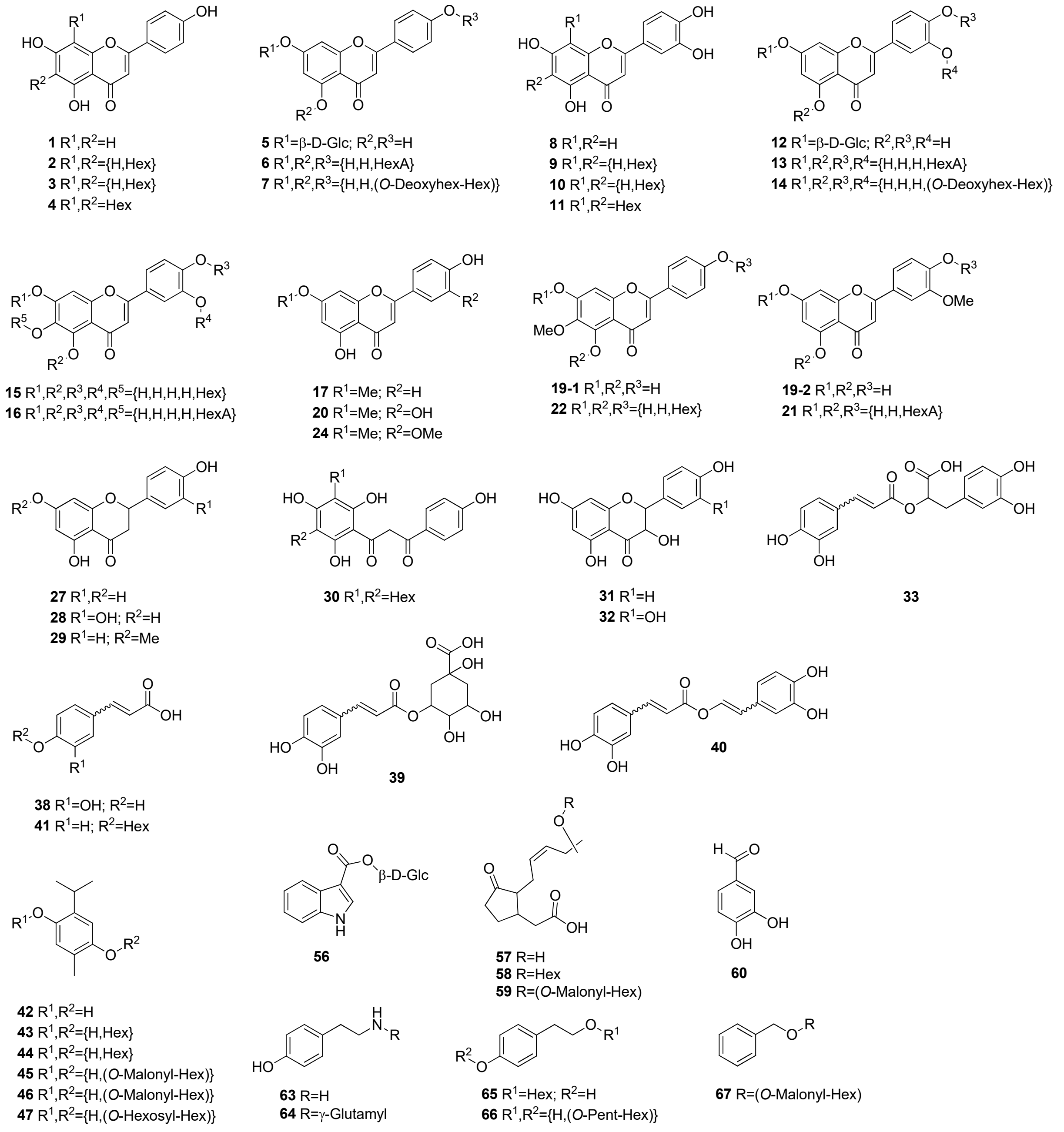


**Figure S2.** Molecular structures of identified and putatively annotated metabolites detected in hydroethanolic leaf extracts of the population Konar Siah. Hex, Hexosyl; HexA, Hexuronosyl; Pent, Pentosyl.

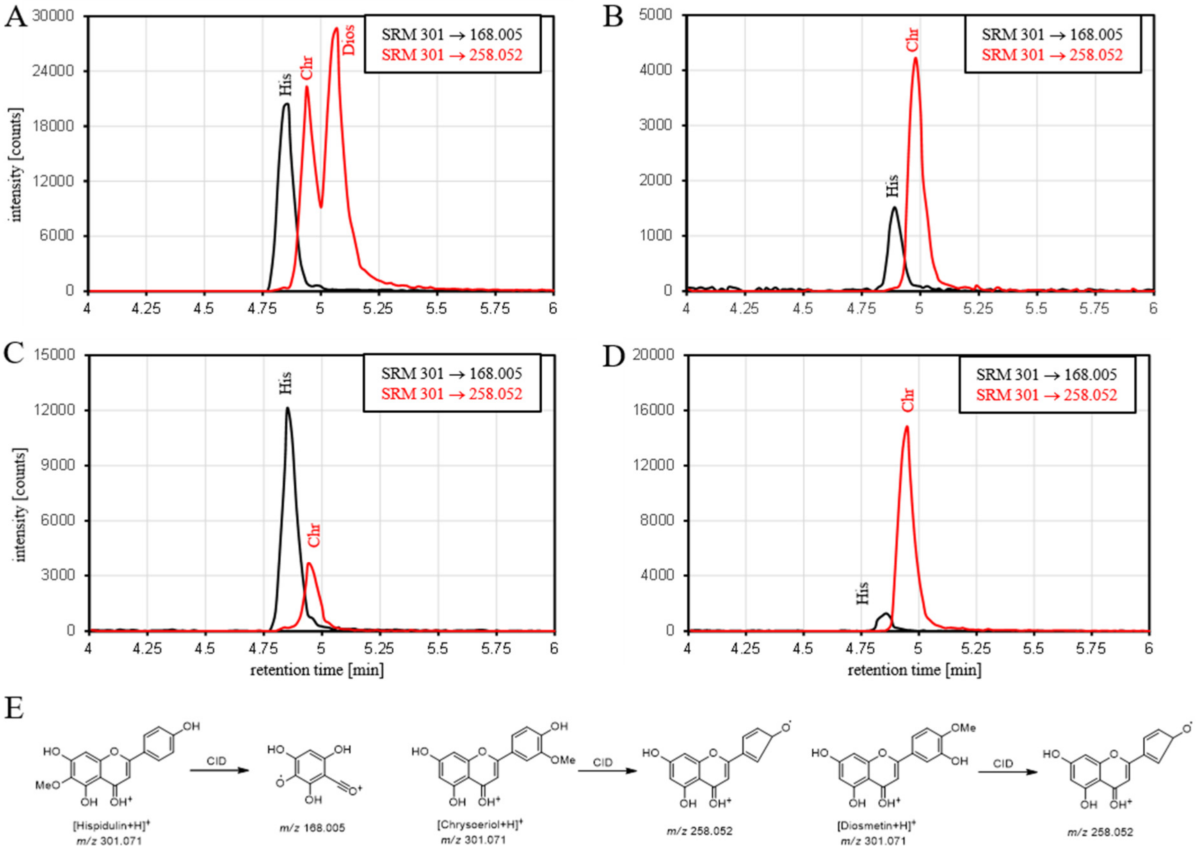


**Figure S3.** Identification of hispidulin and chrysoeriol from hydroethanolic leaf extract of *Z. multiflora*. Single reaction monitoring (SRM) chromatograms obtained from a mixture of authentic hispidulin (His), chrysoeriol (Chr) and diosmetin (Dios) (A). SRM chromatograms obtained from a hydroethanolic leaf extract of *Z. multiflora* (population Konar Siah) (B). SRM chromatograms obtained from a hydroethanolic leaf extract of *Z. multiflora* (population Konar Siah) spiked with authentic hispidulin (C). SRM chromatograms obtained from a hydroethanolic leaf extract of *Z. multiflora* (population Konar Siah) spiked with authentic chrysoeriol (D). Structures of parent and fragment ions used for generation of SRM chromatograms (E). Samples were separated on a Zorbax RRHD Eclipse Plus C18 column (100 × 2.1 mm, 1.8 µm particle size, Agilent Technologies) using 0.1 % (v/v) formic acid in water and 0.05 % (v/v) formic acid in methanol/acetonitrile, 1/1 (v/v) as eluent A and B, respectively. The following binary gradient program at a flow rate of 400 µL min$^{-1}$ was used: 0-10 min, linear from 35 to 55 % B; 10-13 min, isocratic, 95 % B; 13-15 min, isocratic, 35 % B. The column temperature was set at 40 °C. The mass spectrometer was operated in positive ion targeted MS/MS mode. The collision energy was set at 40 V.

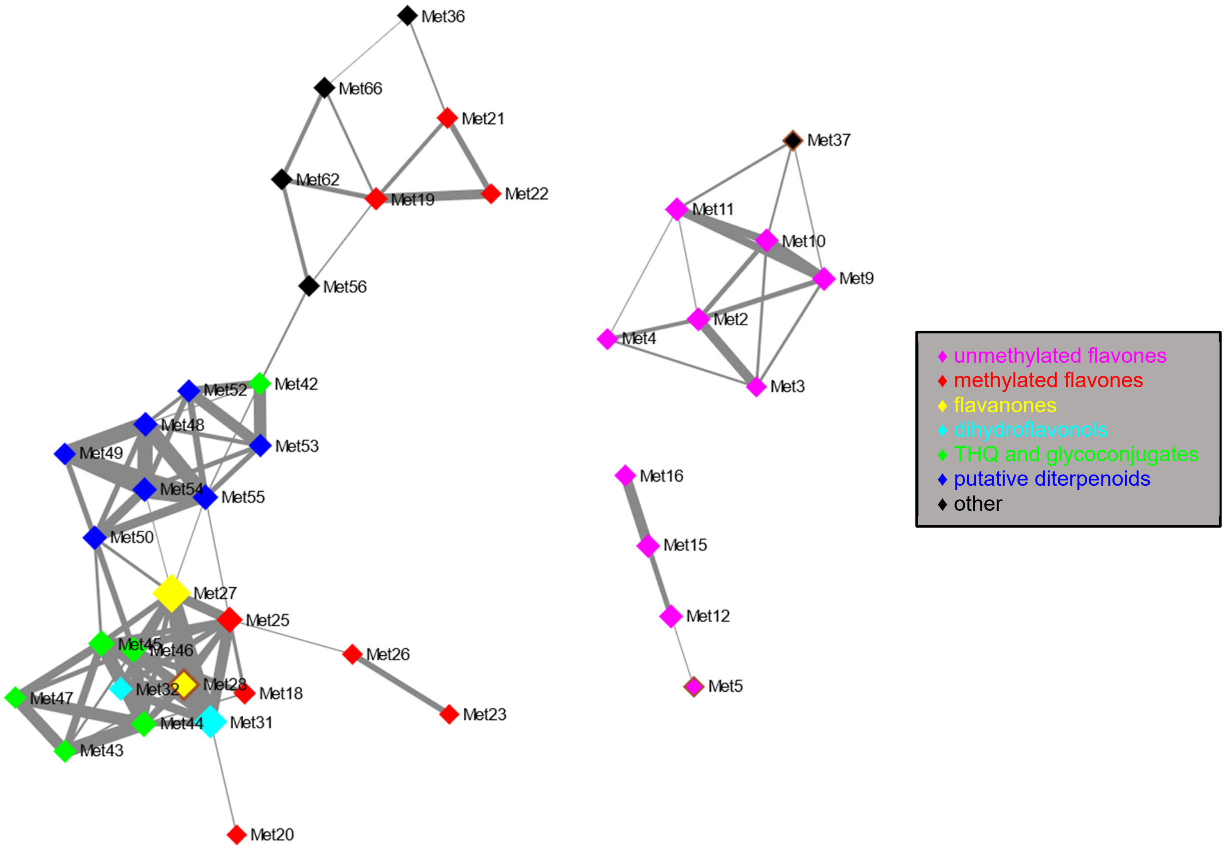


**Figure S4.** Debiased sparse partial correlation network generated from $\log_2$-transformed instrumental drift-corrected peak areas of quantifier ions of metabolites 1-67 detected by UHPLC/DAD/ESI-QTOFMS in hydroethanolic extracts of 42 leaf samples obtained from 14 *Z. multiflora* populations. Subnetworks with at least three nodes (metabolites) are shown. Line thickness indicates correlation strength. Nodes are colored according to metabolite classes.

**Table S2.** Antifungal activities of fractions obtained during bioassay-guided fractionation of a hydroethanolic extract of a pooled *Z. multiflora* leaf sample of the population Konar Siah. Mean growth inhibition rates and standard deviations of three technical replicates are shown. Means in a column not sharing any letter are significantly different (Fisher's least significant difference test, $p \leq 0.05$, Benjamini-Hochberg adjusted). Antifungal assays of fraction 1 to 5 were performed on 9-cm petri dishes using test solutions in ethanol/water, 1/1 (v/v) with a concentration of 10 mg/mL. Due to limited substance quantities, fraction 4-1 to 4-7 were assayed on 4-cm petri dishes at a concentration of 1 mg/mL. $^{*}$Assays not performed due to limited substance quantities.

| **Fraction** | **Inhibition of mycelial growth (%)** | | | | |
|---|---|---|---|---|---|
| | *F. culmorum* | *F. sambucinum* | *B. cinerea* | *A. dauci* | *C. lindemuthianum* |
| Fr. 1 | $17.9 \pm 1.5^{e}$ | $-1.8 \pm 5.9^{d}$ | $13.2 \pm 0.8^{c}$ | $15.9 \pm 1.9^{d}$ | $21.6 \pm 2.1^{d}$ |
| Fr. 2 | $31.9 \pm 3.3^{c}$ | $27.3 \pm 2.3^{c}$ | $26.3 \pm 0.7^{b}$ | $31.1 \pm 3.4^{c}$ | $37.7 \pm 1.4^{c}$ |
| Fr. 3 | $41.1 \pm 1.4^{b}$ | $39.4 \pm 5.3^{b}$ | $48.2 \pm 5.8^{a}$ | $40.2 \pm 3.3^{b}$ | $42.9 \pm 2.9^{b}$ |
| Fr. 4 | $53.1 \pm 2.4^{a}$ | $53.9 \pm 4.3^{a}$ | $48.2 \pm 2.8^{a}$ | $50.2 \pm 1.5^{a}$ | $56.6 \pm 4.8^{a}$ |
| Fr. 5 | $23.9 \pm 2.8^{d}$ | $-5.5 \pm 3.4^{d}$ | $25.1 \pm 1.1^{b}$ | $16.4 \pm 0.4^{d}$ | $19.9 \pm 1.2^{d}$ |
| Fr. 4-1 | $49.8 \pm 4.1^{a}$ | $-^{*}$ | $49.5 \pm 2.5^{a}$ | $-^{*}$ | $27.5 \pm 2.1^{c}$ |
| Fr. 4-2 | $51.9 \pm 5.6^{a}$ | $36.4 \pm 3.9^{c}$ | $32.6 \pm 0.2^{c}$ | $35.6 \pm 3.1^{bc}$ | $44.3 \pm 2.4^{a}$ |
| Fr. 4-3 | $39.7 \pm 2.9^{b}$ | $33.6 \pm 5.1^{cd}$ | $49.2 \pm 3.1^{a}$ | $32.3 \pm 2.9^{c}$ | $27.3 \pm 0.8^{c}$ |
| Fr. 4-4 | $41.8 \pm 4.4^{b}$ | $28.8 \pm 2.7^{d}$ | $20.7 \pm 2.6^{d}$ | $36.1 \pm 3.7^{bc}$ | $35.6 \pm 4.1^{b}$ |
| Fr. 4-5 | $38.1 \pm 3.5^{b}$ | $30.9 \pm 4.3^{cd}$ | $41.8 \pm 2.3^{b}$ | $38.2 \pm 1.2^{b}$ | $40.2 \pm 3.5^{ab}$ |
| Fr. 4-6 | $45.4 \pm 3.7^{ab}$ | $66.2 \pm 2.1^{a}$ | $46.4 \pm 0.1^{a}$ | $55.5 \pm 2.7^{a}$ | $40.1 \pm 2.1^{ab}$ |
| Fr. 4-7 | $50.4 \pm 3.3^{a}$ | $45.9 \pm 2.4^{b}$ | $39.8 \pm 0.3^{b}$ | $33.3 \pm 3.3^{bc}$ | $36.2 \pm 5.7^{b}$ |

**Table S3.** Spearman's correlation coefficients calculated from THQ glycoconjugates abundances and essential oil compounds (*p*-cymene, *γ*-terpinene, thymol and carvacrol). The relative percentage of essential oil compounds in leaves of all 14 populations obtained from the GC-FID peak areas (see Karimi et al., 2020) were used to correlate with the abundances of THQ glycoconjugates in hydroethanolic *Z. multiflora* leaf extracts (n = 42, 14 populations × 3 samples). Statistically significant correlations ($p \leq 0.05$) are marked in bold.

| THQ glycoconjugates | Essential oil compounds | | | |
|---|---|---|---|---|
| | *p*-cymene | *γ*-terpinene | thymol | carvacrol |
| Thymohydroquinone (THQ) | **0.74** | -0.02 | **0.45** | -0.14 |
| THQ *O*-hexoside - isomer#1 | 0.06 | **0.57** | -0.03 | **0.54** |
| THQ *O*-hexoside - isomer#2 | 0.30 | **0.56** | -0.01 | **0.68** |
| THQ *O*-(*O*-malonyl-hexoside) - isomer#1 | **0.54** | **0.50** | 0.05 | **0.63** |
| THQ *O*-(*O*-malonyl-hexoside) - isomer#2 | **0.53** | 0.30 | 0.03 | **0.53** |
| THQ *O*-(*O*-hexosyl-hexoside) | 0.11 | **0.43** | -0.16 | **0.62** |

**Table S4.** General geographic and climatic information of sampled natural habitats of *Z. multiflora*.

| Origin | Code | Province | Latitude (N) | Longitude (E) | Altitude (m a.s.l.) | Temperature (°C) (average annual) | Precipitation (mm/year) |
|---|---|---|---|---|---|---|---|
| Ashkezar | Ash | Yazd | 31° 48' 49" | 54° 00' 26" | 1946 | 21.1 | 40.5 |
| Taft | Taf | Yazd | 31° 42' 26" | 54° 10' | 1697 | 20.3 | 49.8 |
| Arsenjan | Ars | Fars | 29° 53' 49" | 53° 16' 20" | 1865 | 20.3 | 215.2 |
| Darab | Dar | Fars | 28° 44' 27" | 54° 34' 41" | 1276 | 24.3 | 276.4 |
| Fasa | Fas | Fars | 28° 59' 27" | 53° 42' 25" | 1516 | 20.3 | 278.5 |
| Haneshk | Han | Fars | 30° 49' 16" | 53° 18' 19" | 1898 | 14.9 | 180.1 |
| Jandaq | Jan | Esfahan | 33° 57' 44" | 54° 31' 02" | 1235 | 21.5 | 55.9 |
| Siriz | Sir | Kerman | 30° 55' 43" | 55° 57' 01" | 1763 | 20.2 | 107.8 |
| Daarbast | Daa | Hormozgan | 26° 58' 02" | 54° 01' 59" | 1009 | 28.8 | 302.7 |
| Gachooyeh | Gac | Hormozgan | 26° 58' 28" | 53° 58' 06" | 1055 | 28.8 | 302.7 |
| Gezeh | Gez | Hormozgan | 27° 06' 35" | 54° 04' 46" | 731 | 28.8 | 302.7 |
| Hongooyeh | Hon | Hormozgan | 27° 06' 19" | 54° 04' 07" | 820 | 28.8 | 302.7 |
| Kemeshk | Kem | Hormozgan | 27° 03' 13" | 53° 50' 41" | 937 | 28.8 | 302.7 |
| Konar Siah | Kon | Hormozgan | 27° 09' 05" | 53° 57' 04" | 981 | 28.8 | 302.7 |

**Table S5.** Commercial sources of reference compounds used for metabolite identification.

| No. | Name | CAS No. | Supplier |
|---|---|---|---|
| **1** | Apigenin | 520-36-5 | Carl Roth GmbH |
| **5** | Apigenin 7-*O*-β-Glucopyranoside | 578-74-5 | PhytoPlan |
| **8** | Luteolin | 491-70-3 | Extrasynthese |
| **12** | Luteolin 7-*O*- β-Glucopyranoside | 5373-11-5 | Carl Roth GmbH |
| **17** | Genkwanin | 437-64-9 | PhytoLab GmbH - phyproof |
| **19-1** | Hispidulin | 1447-88-7 | PhytoLab GmbH - phyproof |
| **19-2** | Chrysoeriol | 491-71-4 | Extrasynthese |
| **-** | Diosmetin | 520-34-3 | PhytoLab GmbH - phyproof |
| **20** | 7-O-Methylluteolin | 20243-59-8 | Phytolab GmbH - phyproof |
| **-** | Eupatorin | 855-96-9 | PhytoLab GmbH - phyproof |
| **27** | Naringenin | 67604-48-2 | Cayman Chemical |
| **28** | Eriodictyl | 4049-38-1 | PhytoLab GmbH - phyproof |
| **31** | (+)-Aromadendrin | 480-20-6 | Cayman Chemical |
| **32** | (+)-Taxifolin | 480-18-2 | LKT Laboratories |
| **33** | Rosmarinic acid | 20283-92-5 | Carl Roth GmbH |
| **38** | Caffeic acid | 331-39-5 | Carl Roth GmbH |
| **39** | Chlorogenic acid | 327-97-9 | Sigma |
| **-** | Carnosol | 5957-80-2 | PhytoLab GmbH - phyproof |
| **-** | Indole-3-carboxylic acid | 771-50-6 | Sigma-Aldrich |
| **60** | Protocatechualdehyde | 139-85-5 | Aldrich |
| **63** | Tyramine Hydrochloride | 60-19-5 | Sigma |
